# Genetic and clinical characteristics of patients with lipoprotein lipase deficiency from Slovenia and Pakistan: case series and systematic literature review

**DOI:** 10.3389/fendo.2024.1387419

**Published:** 2024-06-07

**Authors:** Quratul Ain, Matija Cevc, Tatiana Marusic, Jaka Sikonja, Fouzia Sadiq, Ursa Sustar, Matej Mlinaric, Jernej Kovac, Hijab Batool, Mohammad Iqbal Khan, Katarina Trebusak Podkrajsek, Barbara Jenko Bizjan, Tadej Battelino, Zlatko Fras, Muhammad Ajmal, Urh Groselj

**Affiliations:** ^1^ Translational Genomics Laboratory, Department of Biosciences, COMSATS University Islamabad, Islamabad, Pakistan; ^2^ Directorate of Research, Shifa Tameer-e-Millat University, Islamabad, Pakistan; ^3^ Division of Medicine, Centre for Preventive Cardiology, University Medical Centre Ljubljana, Ljubljana, Slovenia; ^4^ Department of Endocrinology, Diabetes, and Metabolic Diseases, University Children’s Hospital, University Medical Centre Ljubljana, Ljubljana, Slovenia; ^5^ Department of Endocrinology, Diabetes and Metabolic Diseases, Division of Medicine, University Medical Centre Ljubljana, Ljubljana, Slovenia; ^6^ Faculty of Medicine, University of Ljubljana, Ljubljana, Slovenia; ^7^ Department of Clinical Chemistry and Immunology, Chughtai Institute of Pathology, Lahore, Pakistan; ^8^ Department of Vascular Surgery, Shifa International Hospital, Islamabad, Pakistan

**Keywords:** hypertriglyceridemia, lipoprotein lipase, LPL, lipoprotein lipase deficiency, pancreatitis, case series

## Abstract

**Introduction:**

Hypertriglyceridemia (HTG) is a complex disorder caused by genetic and environmental factors that frequently results from loss-of-function variants in the gene encoding lipoprotein lipase (LPL). Heterozygous patients have a range of symptoms, while homozygous LPL deficiency presents with severe symptoms including acute pancreatitis, xanthomas, and lipemia retinalis.

**Methods:**

We described the clinical characteristics of three Slovenian patients (an 8-year-old female, an 18-year-old man, and a 57-year-old female) and one Pakistani patient (a 59-year-old male) with LPL deficiency. We performed next-generation sequencing (NGS) targeting all coding exons and intron-exon boundaries of the *LPL* gene, and Sanger sequencing for variant confirmation. In addition, we performed a systematic literature review of all cases with three identified variants and described their clinical characteristics.

**Results:**

Two Slovenian patients with a heterozygous pathogenic variant NM_000237.3:c.984G>T (p.Met328Ile) were diagnosed within the first three years of life and had triglyceride (TG) values of 16 and 20 mmol/L. An asymptomatic Pakistani patient with TG values of 36.8 mmol/L until the age of 44 years, was identified as heterozygous for a pathogenic variant NM_000237.3:c.724G>A (p.Asp242Asn). His TG levels dropped to 12.7 mmol/L on dietary modifications and by using fibrates. A Slovenian patient who first suffered from pancreatitis at the age of 18 years with a TG value of 34 mmol/L was found to be homozygous for NM_000237.3:c.337T>C (p.Trp113Arg).

**Conclusions:**

Patients with LPL deficiency had high TG levels at diagnosis. Homozygous patients had worse outcomes. Good diet and medication compliance can reduce severity.

## 1 Introduction

Hypertriglyceridemia (HTG) is a multifaceted lipid metabolism disorder, influenced by genetic and environmental factors. It is estimated that approximately 30% of the global population is affected by HTG i.e. triglycerides (TG) more than 150 mg/dL, with men (37%) being more commonly affected than women (23%) (1). TG levels between 150–199 mg/dL are classified as mild HTG, while levels ranging from 200–999 mg/dL indicate moderate HTG. Severe HTG is characterized by levels between 1000–1999 mg/dL, and very severe HTG is defined as levels ≥2000 mg/dL (2). While only a small percentage (1.1%) of individuals exhibit biallelic or homozygous monogenic HTG, also known as familial chylomicronemia (FCS), the majority of cases (46.3%) are attributed to polygenic HTG while the remaining 52.6% HTG cases are genetically unidentified (3). The majority of FCS cases (around 95%) are associated with specific pathogenic variants of the lipoprotein lipase (*LPL*) gene, and 5% of FCS cases are linked to variants in four other genes (*APOC, APOA5, LMF1*, and *GPIHBP1*), encoding proteins that modulate the activity of LPL (4, 5) Multifactorial chylomicronemia syndrome (MCS) is a common cause of severe hypertriglyceridemia, linked to risks of acute pancreatitis, cardiovascular issues, and non-alcoholic steatohepatitis. Moulin et al. (6) have proposed a diagnostic algorithm to differentiate FCS from MCS. FCS score is calculated based upon certain clinical features of patients i.e., the severe elevation of plasma TGs, resistance to standard TG-lowering therapies, young age at onset, absence of secondary factors, and a history of acute pancreatitis episodes. If the score is 10 or higher, FCS is highly probable; if it’s 9 or lower, FCS diagnosis is unlikely, and if it’s 8 or lower, FCS is highly unlikely (6).

LPL is located on vascular endothelial cells in adipose tissue, the heart, and muscles, hydrolyzing TG-rich lipoproteins, such as chylomicrons and very low-density lipoproteins (VLDL), facilitating their clearance from the blood (7). To date, 250 distinct variants have been reported in the *LPL* gene, among these the most common being missense/nonsense pathogenic variants (8, 9). Diagnosis of LPL deficiency can be challenging due to variations in biochemical parameters and often overlapping phenotypes with other etiologically different HTGs (10).

LPL deficiency (OMIM #238600) is characterised by the accumulation of TG-rich lipoproteins (11). Individuals with partial LPL deficiency, due to heterozygous variants, have reduced LPL enzyme activity. This leads to elevated postprandial TG levels and an increased risk of pancreatitis and cardiovascular disease. While, patients with homozygous *LPL* variants experience complete LPL deficiency, resulting in no LPL enzyme activity. This leads to severe HTG and associated complications such as pancreatitis, abdominal pain, eruptive xanthomas, and hepatosplenomegaly, which are unresponsive to standard treatments (6, 12, 13).

The primary objective of our current study is to comprehensively characterise the genetic and clinical features of four patients diagnosed with LPL deficiency, from both Slovenia and Pakistan. This will provide insight into how these variants may contribute to the development and progression of LPL deficiency. By comparing the clinical profiles of these patients, we seek to identify influences on the disease presentation, severity, and associated complications. Additionally, we conducted a systematic review of the existing literature to further explore the clinical characteristics of patients, associated with identified *LPL* genetic variants, in our study. We aim to improve the understanding of the intricate interplay between genetics and clinical manifestations of LPL deficiency, ultimately contributing to improved disease management.

## 2 Materials and methods

### 2.1 Study design and cohort description

In this retrospective observational study, we examined the medical health records of four unrelated patients diagnosed with HTG. Patients 1, 2, and 4 are an 8-year-old female and an 18-year-old male, and 57 years old female were managed at the University Medical Centre Ljubljana, Slovenia, while patient 3 aged 59 years old male was managed at a tertiary care hospital in Pakistan.

### 2.2 Compliance with ethics guidelines

The study was approved by the National Medical Ethics Committee of the Republic of Slovenia (number 0120–14/2017/5, and 0120–273/2019/9) and by the institutional review board and ethics Committee of Shifa Tameer-e-Millat University Islamabad, Pakistan (033–523-2019). The principles of the Declaration of Helsinki were followed in this study. All adult patients or their caregivers for child patients provided written informed consent before inclusion in the study.

### 2.3 Genetic testing

Genetic analysis was conducted at the University Children’s Hospital, Ljubljana, Slovenia, following a previously established protocol in Slovenia (14).

Genomic DNA was isolated from a venous blood sample using a Flexigene kit (Qiagen, Germany). To target the dyslipidemia-associated genes’ coding regions and intron-exon borders, xGen^®^ Lockdown^®^ NGS Probes were employed. Next-generation sequencing (NGS) was performed using the MiSeq Reagent Kit (Illumina, United States) on a MiSeq sequencer, following the manufacturer’s protocol.

The following genes were included in the analysis: *ABCA1, ABCG5, ABCG8, ALMS1, APOA1, APOA5, APOB, APOC2, APOC3, APOE, CREB3L3, GPIHBP1, LDLR, LDLRAP1, LIPA, LMF1, LPL, and PCSK9*. candidate pathogenic variants identified through NGS following previously described protocol (14) were validated using Sanger sequencing.

### 2.4 Systematic literature review

**Figure 1:**
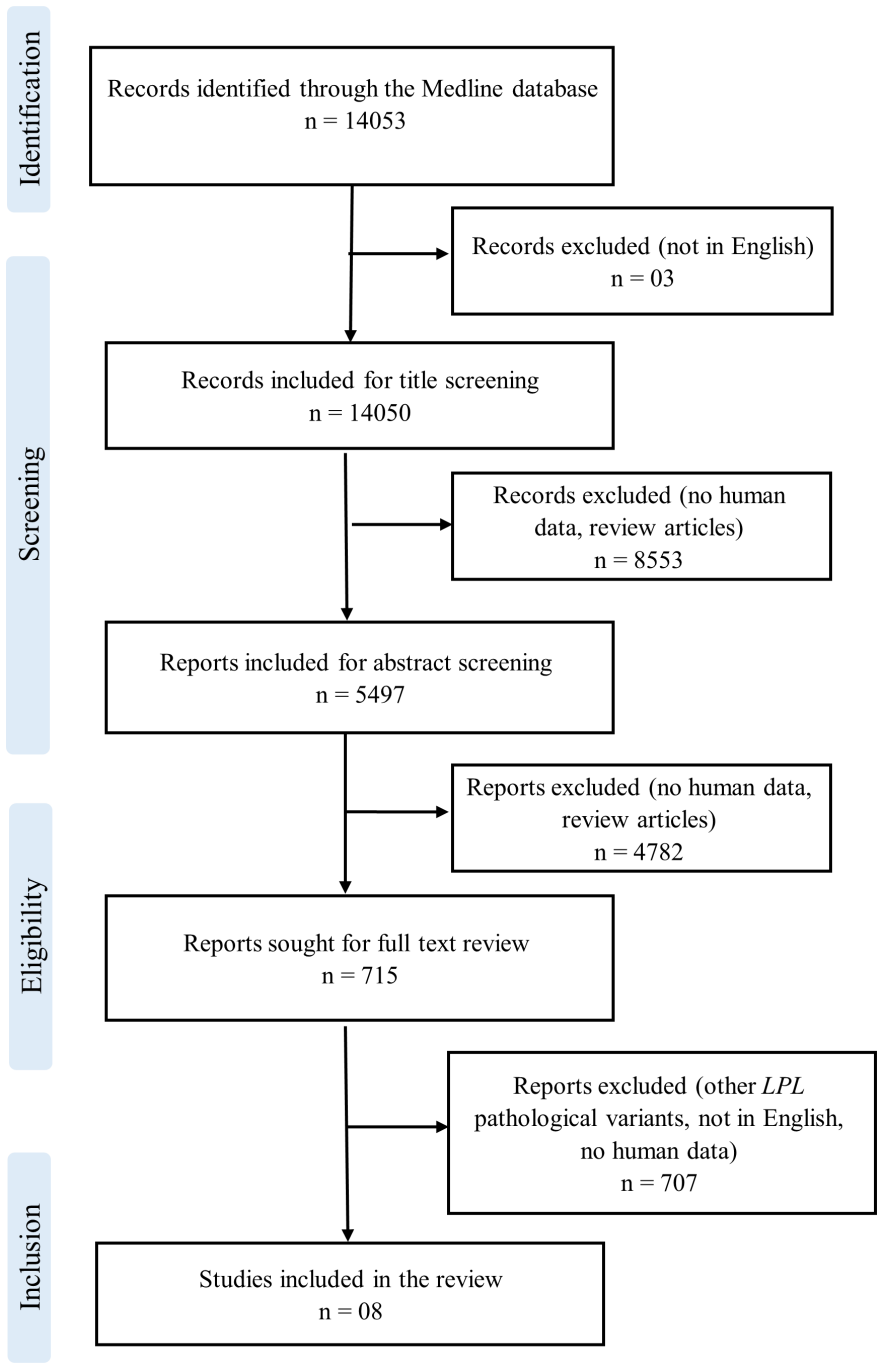
PRISMA flow diagram for systematic literature review.

Following the PRISMA reporting guidelines, we performed a systematic review to summarize the data about all patients with either one of the three *LPL* variants (c.984G>T, c.337T>C, and c.724G>A) from our study on 24^th^ March 2023. Our search focused on the Medline database, using the search term “lipoprotein lipase,” which resulted in the identification of 14,053 records. To ensure the relevance and quality of the included studies, two independent reviewers carefully reviewed the titles, abstracts, and full-length text of the identified articles. These inclusion criteria comprised human-based studies published in English, specifically reporting on *LPL* variants related to our research, and providing clinical data, particularly TGs, along with information on pathogenic variants (**Figure 1**). From this comprehensive screening process, we selected only eight records that met our predetermined inclusion criteria.

In addition, we also searched through HGMD and Franklin databases to identify additional reports that were not found in Pubmed.

## 3 Results

Patient 1 was incidentally discovered in a female at the age of 3 years, during systemic bacterial infection, with her initial recorded TG level being 16.8 mmol/L, indicative of primary HTG. The patient was found to have a heterozygous pathogenic variant NM_000237.3:c.984G>T (p.Met328Ile) in exon 6 of the *LPL* gene. Following dietary modifications, including a low-fat diet, omega-3 fatty acid supplementation, and avoidance of high-carbohydrate foods, patient 1’s TG levels significantly decreased to 1.9 mmol/L at the last check-up. **Table 1** provides a summary of patients’ characteristics. The patient had no family history of dyslipidemia or cardiovascular disease.

**Table 1:** Demographic and clinical details of presented patients.

Pt.	Age (years)	Sex	Age at diagnosis (years)	Clinical diagnosis*	TG at first presentation (mmol/L)	TG at last follow-up (mmol/L)	Fasting TG >10. (3 times) (mmol/L)	History of TG <2 (mmol/L)	History of AP	Recurrent Abdominal Pain	FCH	Secondary factors	FCS**	*LPL* (NM_000237.3)
1	8	F	3	Severe HTG	16.8	1.9	Yes	Yes	No	No	Yes	No	FCS very unlikely (Score=4)	Heterozygous. c.984G>T
2	18	M	At birth	Severe HTG	20.0	8.0	Yes	No	No	No	No	No	FCS very unlikely (Score=8)	Heterozygous c.984G>T
3	59	M	44	Very severe HTG	36.8	2.7	Yes	No	No	Yes	No	Yes	FCS very unlikely (Score=8)	Heterozygous c.724G>A
4	57	F	18	Very severe HTG	34.0	11.0	Yes	No	Yes	Yes	No	No	FCS very likely (Score=13)	Homozygous c.337T>C

Pt., patient; F, Female; M, Male; AP, Acute pancreatitis; FCH, Familial combined hyperlipidemia.

*clinical diagnosis is based upon The Endocrine Society guidelines (2).

**FCS diagnosis is based on a score described by (6).

Patient 2 was a male was born at 37 weeks with a body weight of 2930 g and required mechanical support due to meconium aspiration. His plasma TG levels were 20 mmol/L during the neonatal period, and genetic analysis of the patient revealed a heterozygous pathogenic variant in the *LPL* gene, specifically NM_000237.3:c.984G>T (p.Met328Ile). Dietary restrictions including breast milk were maintained, resulting in a last recorded TG level of 8 mmol/L. The patient’s mother also exhibited high TG levels (11 mmol/L). The calculated FCS score was 8 (**Table 1**).

Patient 3, a 44-years male was diagnosed with HTG with a TG value of 36.8 mmol/L. The patient underwent genetic testing, which confirmed a heterozygous pathogenic variant in exon 5 of *LPL*: NM_000273:c.724G>A (p.Asp242Asn). Treatment with gemfibrozil 600 mg, and diet significantly reduced TG levels to 2.7 mmol/L. The patient also had type 2 diabetes and chronic kidney disease. It was noted that patient 3’s brothers also exhibited TG levels above 12 mmol/L.

Patient 4, a female, experienced her first episode of pancreatitis at 18 years old. Upon investigation, a TG level of 34 mmol/L was revealed. The FCS score of the patient was 13. Genetic testing of the patient revealed a homozygous variant in *LPL* NM_000237.3:c.337T>C (p.Trp113Arg). Despite various attempts, patient 4 continued to experience persistent HTG due to non-adherence to a strict low-fat diet. This led to seven episodes of AP and the development of multiple pancreatic cysts, which required surgical intervention. Treatment with fenofibrate and omega-3 fatty acids, along with the adoption of an appropriate diet, significantly lowered TG levels to 11 mmol/L at the last visit. Her father also suffered from HTG and experienced a heart attack at the age of 65 years.

### 3.1 Comparison of cases from our study and from systematic literature review

We have comprehensively reviewed previous studies on the *LPL* c.984G>T pathogenic variant, as presented in **Table 2**. Homozygous *LPL* variants resulted in elevated TG levels (65.6 mmol/L) and severe clinical complications, such as hepatosplenomegaly and AP, whereas heterozygous patients showed an average TG value of 11.4 mmol/L. On the other hand, patients with compound heterozygous (CHET) variants had the highest average TG levels (163.7 mmol/L).

**Table 2:** Previously reported individuals with pathogenic *LPL* variants c.984G>T with their clinical details.

Patient ID	Nationality	Sex	Age at diagnosis (Years)	Zygosity	TG at presentation mmol/L (mg/dL)	TG management Technique	TG decreased (%)	Eruptive xanthomatosis	HSM	AP	Consang.	Reference
1	Slovenian	F	3	HET	16.8 (1488)	low-fat diet, omega-3 fatty acid supplementation	89	No	No	No	No	This study 2024
2	Slovenian	M	At birth	HET	20.0 (1771)	low-fat milk	60	No	No	No	No	This study 2024
3	Italian	M	78	HOM	33.9 (3000)	NA	NA	No	No	Yes	Yes	(15)
4	Italian	F	1 month	HOM	139.9 (12390)	NA	NA	No	No	No	NA	(16)
5	Italian	M	1 month	HOM	23.2 (2054)	NA	NA	No	No	No	NA	(16)
6	Italian	F	9months	CHET	109.5 (9698)	NA	NA	No	No	No	NA	(16)
7	Italian	M	10	HET	7.9 (700.6)	NA	NA	No	No	No	NA	(16)
8	Italian	F	30	HET	1.0 (84.1)	NA	NA	No	No	No	NA	(16)
9	Italian	F	0.5 month	CHET	218.0 (19308.3)	low-fat formula milk, Monogen	97%	NA	NA	NA	NA	(17)

F, Female; M, Male; HSM, Hepatosplenomegaly; AP, Acute pancreatitis; Consang, Consanguinity; HET, Heterozygous; HOM, Homozygous; CHET, Compound Heterozygous; NA, Not available.

Homozygous patients with pathogenic *LPL* variants c.337T>C had a higher likelihood of experiencing acute pancreatitis, while compound heterozygous and heterozygous patients seemed to experience milder or no complications. The average TG values were highest for homozygous patients (161.3 mmol/L), followed by heterozygous patients (48.4 mmol/L). The only reported case of CHET patients with c.337T>C variant had a TG level of 39.7 mmol/L (**Table 3**).

**Table 3:** Previously reported individuals with pathogenic *LPL* variants c.337T>C with their clinical details.

Patient ID	Nationality	Sex	Age at diagnosis (Years)	Zygosity	TG at presentation mmol/L (mg/dL)	TG management Technique	TG decreased (%)	Eruptive xanthomatosis	HSM	AP	Consang.	Reference
1	Slovenian	F	18	HOM	34.0 (3011.4)	fenofibrate and omega-3 fatty acids	68	No	No	Yes	NA	This study 2024
2	Austrian	NP	19	HOM	338.7 (30000.0)	Low-fat diet, medium-chain FAs	20	No	No	Yes	NA	(18)
3	Austrian	F	14	HOM	111.3 (9859.0)	very low-fat diet, Maltodextrin, Omacor, MCT oil, 6g essential FA	97	No	Yes	Yes	NA	(19)
4	Swedish	F	49	CHET	39.7 (3516.2)	very low-fat diet	34	No	No	Yes	Yes	(20)
5	Italian	M	1 month	HET	76.8 (6800.0)	low fat formula milk	93	No	No	No	No	(21)
6	Spanish	NP	NP	HET	>20 (>1771.4)	NA	NA	NA	NA	NA	NA	(22)

F, Female; M, Male; HSM, Hepatosplenomegaly; AP, Acute pancreatitis; Consang, Consanguinity; HET, Heterozygous; HOM, Homozygous; CHET, Compound Heterozygous; NA, Not provided.

Comprehensive analysis of the baseline TG levels at the time of diagnosis and TG levels after implementing TG management techniques showed a significant reduction in the TG levels among all of the patients. The study further revealed that the use of low-fat food in adult patients and low-fat milk in infants led to a significant decrease in TG levels.

However, we could not find enough studies focused on the *LPL* variant c.724G>A. A study reporting the variant in the Argentinian population was identified, but it didn’t meet our review criteria (23).

## 4 Discussion

The present study reports four patients who had pathogenic variants in the *LPL* gene, encoding LPL, a pivotal enzyme in the regulation of TG metabolism (24). Any pathogenic variation in the *LPL* gene results in the reduction or absence of LPL activity, resulting in higher TG levels in plasma, which in turn leads to the accumulation of TGs in various tissues resulting in pancreatitis, eruptive xanthomas, and hepatosplenomegaly (25). Severe and very severe HTG increase the risk of pancreatitis, while mild or moderate HTG may be a risk factor for cardiovascular disease (2).

We have reported two Slovenian patients with c.984G>T (p.Met328Ile), one Slovenian patient with c.337T>C (p.Trp113Arg), and one Pakistani patient with c.724G>A (p.Asp242Asn) variants in the *LPL* gene. All patients included in this study presented with HTG when diagnosed. Similar results were obtained in different studies conducted, where *LPL* pathogenic variants were associated with HTG (9, 26–30). Severe HTG is the third most common reason for developing AP following gallstones and alcohol abuse (31). There is no exact TG threshold level that causes AP, but the risk steadily rises as TG levels increase (32). Patient 4 from our study with c.337T>C (p.Trp113Arg) homozygous *LPL* variant presented with recurrent pancreatitis. Patient 4 exhibited a severe phenotype attributed to FCS, confirmed by genetic testing and a high FCS score. Similar to our findings various studies have been reported to link this pathogenic variant with pancreatitis (19–21).

HTG and inadequate glycemic control elevate the likelihood of chronic kidney disease (CKD) in individuals with type 2 diabetes mellitus (33). The presence of nephrotic syndrome is associated with a significant reduction in both the abundance and activity of the LPL protein (34). Patient 3 had type II diabetes mellitus and chronic kidney disease, along with HTG, indicating the MCS. An analysis of 18 genes in our NGS panel did not identify any other pathogenic or VUS variants. However, considering the severity of HTG and the coexistence of diabetes and CKD, it is likely that additional genetic variants in other genes may be contributing to the patient’s condition. It is recommended to conduct further genetic testing beyond the current gene panel to identify these potential contributors.

For LPL deficiency patients, diet change, and lifestyle modifications are considered as the first line of treatment. Medications like fibrates are prescribed which increase lipoprotein lipase activity, reducing VLDL levels in the liver (2, 35, 36). Keeping plasma TG below 11.30 mmol/L can help prevent serious complications like severe pancreatitis, which ultimately reduces the high mortality and morbidity rate. In our patients, the implementation of a fat-free diet, lifestyle modification, and medication resulted in a sharp decline in TG levels. Unfortunately, due to resource limitations and the scope of our study, we could not include parental data, which would have provided valuable insights into genetic inheritance patterns. NGS has certain limitations, such as missing large deletions, duplications, or deep intronic mutations, so there is the possibility that one of our heterozygous patients might have an undetected variant, resulting in compound heterozygosity. We analysed *LPL* variants in Pakistani and Slovenian, complete, and partial LPL deficient populations, providing a deeper understanding of genotype-phenotype relationships for better diagnostics and treatment options. The cases reported in this manuscript contribute to the knowledge, base for precision medicine in the future. In summary, our research reveals that pathogenic variations in the *LPL* gene are clinically relevant and linked to the severity of HTG.

## Data availability statement

The original contributions presented in the study are included in the article/supplementary material, further inquiries can be directed to the corresponding author/s.

## Ethics statement

The study was approved by the National Medical Ethics Committee of the Republic of Slovenia (number 0120-94 14/2017/5, and 0120-273/2019/9) and by the institutional review board and ethics Committee of Shifa Tameer-95 e-Millat University Islamabad, Pakistan (033-523-2019). The studies were conducted in accordance with the local legislation and institutional requirements. Written informed consent for participation in this study was provided by the participants’ legal guardians/next of kin.

## Author contributions

QA: Writing – review & editing, Writing – original draft. MC: Writing – review & editing, Writing – original draft. TM: Writing – review & editing, Writing – original draft. JS: Writing – review & editing, Writing – original draft. FS: Writing – review & editing, Writing – original draft, Visualization, Validation, Supervision, Software, Resources, Project administration, Methodology, Investigation, Funding acquisition, Formal Analysis, Data curation, Conceptualization. US: Writing – review & editing, Writing – original draft. MM: Writing – review & editing, Writing – original draft, Data curation. JK: Writing – review & editing, Investigation. HB: Writing – review & editing, Data curation. MK: Writing – review & editing, Supervision, Data curation. KT: Writing – review & editing, Supervision, Resources. BB: Writing – review & editing, Supervision, Methodology. TB: Writing – review & editing, Software, Investigation. ZF: Writing – review & editing, Investigation, Formal Analysis. MA: Writing – review & editing, Supervision. UG: Writing – review & editing, Writing – original draft, Visualization, Validation, Supervision, Software, Resources, Project administration, Methodology, Investigation, Funding acquisition, Formal Analysis, Data curation, Conceptualization.
